# Magnesium sulfate treatment for acute severe asthma in adults—a systematic review and meta-analysis

**DOI:** 10.3389/falgy.2023.1211949

**Published:** 2023-07-28

**Authors:** Alma Holm Rovsing, Osman Savran, Charlotte Suppli Ulrik

**Affiliations:** ^1^ Department of Respiratory Medicine, Copenhagen University Hospital-Hvidovre, Hvidovre, Denmark; ^2^ Institute of Clinical Medicine, University of Copenhagen, Copenhagen, Denmark

**Keywords:** MgSO4, intravenous, nebulized, acute asthma, systematic review, meta-analysis, PEF, FEV_1_

## Abstract

**Introduction:**

Add-on magnesium sulfate (MgSO4) for refractory asthma exacerbation has been much debated. The aim of this review and meta-analysis is, therefore, to provide an update on the current evidence for the efficacy of MgSO4 in exacerbations of asthma in adults refractory to standard of care treatment.

**Methods:**

A systematic review was performed in accordance with the PRISMA guidelines. The search was performed in the PubMed database (updated April 2023). For the meta-analysis, a random-effects model was applied using the metaphor package for RStudio (RStudio, Inc.).

**Results:**

A total of 17 randomized controlled trials were included. Three of the nine studies addressing treatment with intravenous (IV) MgSO4 found a significant effect on lung function compared to placebo. Of the eight studies investigating hospital admission rate, only two found a significant effect of MgSO4. Six of the nine studies investigating treatment with nebulized MgSO4 compared to placebo found a favorable effect on forced expiratory volume in 1. second (FEV_1_) and peak expiratory flow rate (PEF). Only two of the five studies investigating the effect on hospital admission rate found an effect of MgSO4. Comparing effect sizes in a meta-analysis revealed a greater effect on PEF in asthma patients treated with nebulized MgSO4 (MD, 23.57; 95% CI, −2.48 to 49.62, *p* < 0.01) compared to placebo. The analysis of patients treated with i.v. MgSO4 compared to placebo showed no statistically significant difference (MD, 5.49; 95% CI, −18.67 to 29.65, *p* = 0.10).

**Conclusion:**

Up to two out of three studies revealed an effect of MgSO4 treatment for asthma exacerbation when assessed by FEV_1_/PEF, but fewer studies were positive for the outcome of hospital admissions.

## Introduction

Asthma is a chronic inflammatory airway disease that even in patients with mild disease is associated with periodically severe worsening, also referred to as exacerbations (1). While mild-to-moderate exacerbations may be managed in primary care, more severe exacerbations often require management in the ER and/or hospital admission.

In Denmark, a country of 5.8 million inhabitants, asthma exacerbations result in approximately 1,400 ER visits and 6,300 hospital admissions each year (2). The standard of care for patients with severe acute exacerbations managed in hospitals comprises supplemental oxygen, high doses of nebulized short-acting β2 agonists (SABA) in combination with a short-acting muscarinic antagonist (SAMA), and systemic corticosteroids (3, 4). Despite the initial treatment in the ER, some patients do not improve sufficiently and, therefore, require admission for further treatment. In very severe acute exacerbations of asthma refractory to standard treatment, the patient may need to be transferred to the intensive care unit (ICU), where intubation and mechanical ventilation may be needed. In the United States, about 10% of patients admitted with asthma exacerbations were reported to require transferal to the ICU in the year 2,000 (5), but the proportion differs between countries at least partly due to differences in referral criteria.

In severe refractory acute asthma, a number of guidelines recommend add-on intravenous infusion and/or nebulized magnesium sulfate (MgSO4) (3, 4), but in contrast to the standard treatment of asthma exacerbations, the clinical effect of MgSO4 has been much debated.

The aim of this review and meta-analysis is to provide an update on the present evidence for the efficacy of MgSO4 in the treatment of acute refractory asthma in adults.

## Methods

This review and meta-analysis were carried out in accordance with the PRISMA statement (6).

A systematic search was performed in the PubMed, Medline, and Embase databases and updated in April 2023. The strategy was to identify all RCTs addressing the treatment of acute asthma with MgSO4. The search algorithm consisted of whole words (acute asthma AND magnesium sulfate) combined with the MeSH terms (“asthma” AND “magnesium sulfate”). All of the records were systematically reviewed by all the authors using Covidence; first on the title/abstract level, then on the full-text level. All conflicts were handled by at least two of the authors, who discussed why or why not to include the study of conflict.

Publications were included in the present review provided they fulfilled the following criteria: (1) addressed treatment of acute asthma in adults (≥15 years) with magnesium sulfate (i.v./nebulized), (2) RCT, (3) published in 1990 or later, and that they did not fulfil the following criteria: (1) non-RCT, (2) addressing treatment of asthma in children, and (3) non-English publication.

The main outcomes of focus were FEV_1_, PEF, and hospital admission/discharge.

To avoid missing any relevant studies, all reference lists of the included studies and previous reviews were scanned for additional studies potentially fulfilling the criteria for inclusion in the present review.

After including all relevant studies, a risk-of-bias analysis was performed using the Cochrane risk-of-bias tool for randomized trials (7).

A meta-analysis of the studies fulfilling the criteria was conducted. Studies were included in the meta-analysis if they provided relevant data on peak expiratory flow (PEF). Most studies provided PEF at the baseline and at the end of the study period, whereas studies had missing data on FEV_1_ and variation in definition of hospital admissions, which made PEF the preferred variable for inclusion in a meta-analysis of the study findings. Duration of treatment with either nebulized or intravenous magnesium or placebo until the end of the study period differed between studies. Some studies provided treatment durations ranging from one to four hours, while others only provided a final PEF before discharge. We calculated the mean difference (MD) with 95% confidence intervals to assess the final difference in PEF between patients and placebo. We assessed heterogeneity in the included trials and considered a *p*-value threshold of 0.1 or less for the test of heterogeneity or less as statistically significant. Random-effects models were used for the meta-analysis if statistical significance was present. A random-effects model analysis was carried out using the metafor package of RStudio Version 1.2.5001 2009–2019 RStudio, Inc.

The present study was a systematic review and meta-analysis and, therefore, approval from the scientific ethical committee and the Danish Medicines Agency was not required.

## Results

**Figure 1:**
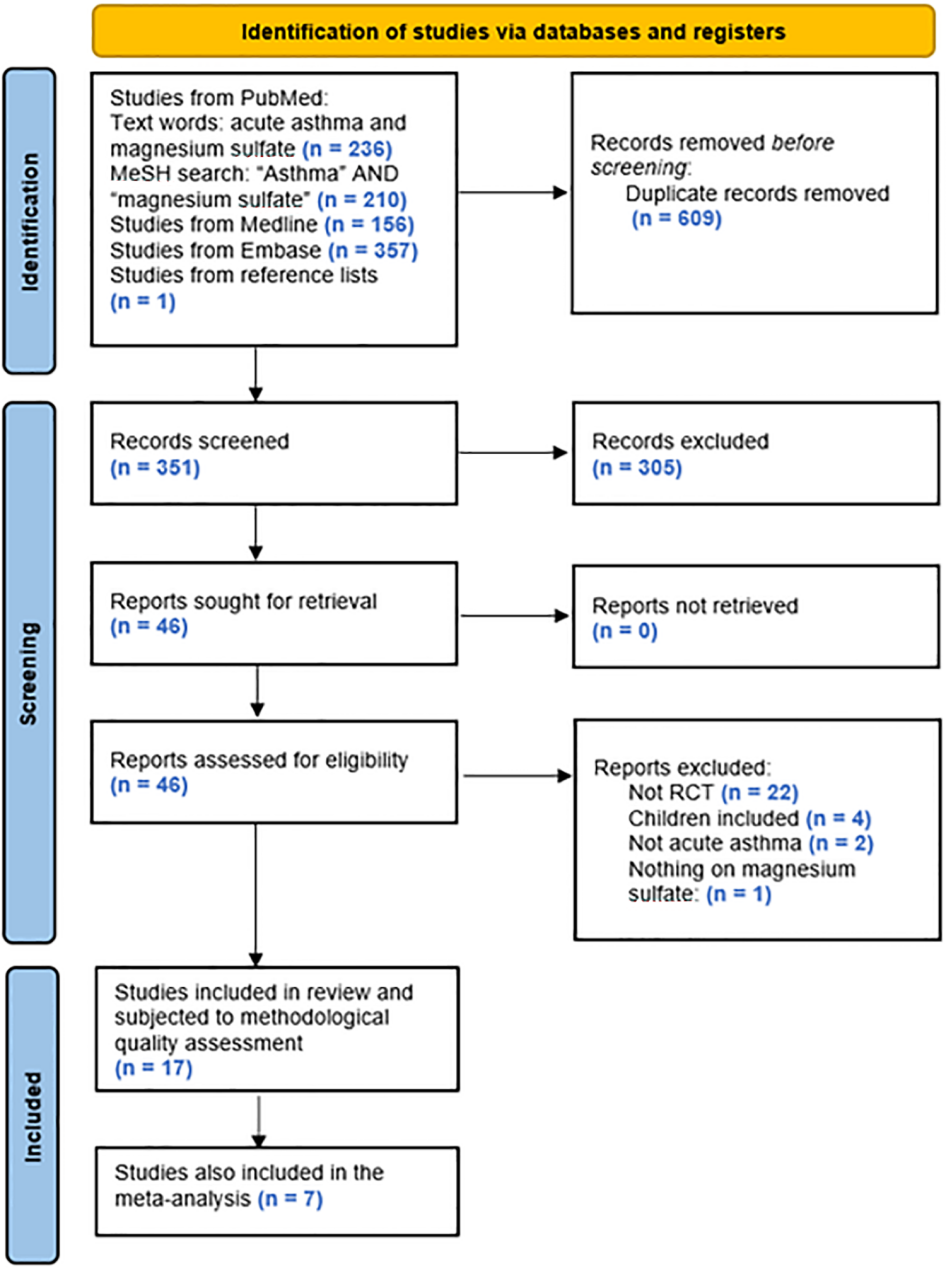
PRISMA 2020 flow diagram.

The search algorithm provided 236, 210, 156, and 357 hits, respectively, of which 16 studies fulfilled the predefined criteria and were included in the present review. Based on the additional search described, 1 further study fulfilling the criteria was identified and added, leading to a total of 17 studies included (Figure 1).

Of the 17 studies included, 8 assessed i.v. MgSO4 treatment, 8 investigated treatment with nebulized MgSO4, and 1 assessed both i.v. and nebulized MgSO4 treatment.

Apart from one study, all studies investigating hospital admission provided exact criteria for hospital admission (8). In the former study, however, the clinician responsible for the decision was blinded to treatment allocation and may therefore not be considered biased (8).

Only one study mentioned that asthma diagnosis had previously been objectively verified (bronchodilator reversibility) (9). The largest study included did not find any significant difference between placebo and either i.v. or nebulized MgSO4 with regard to hospital admission rate or improvement in PEF%predicted (10).

Table 1 summarizes the main characteristics of the included studies.

**Table 1:** Overview of the studies on MgSO4, comparing designs, outcome measurements, study populations, different treatments, and results.

Study	Design	Objective	Outcomes	Patients	Treatment	Result
Green (11), 1992, California, United States	RCT	I.v. MgSO4 as adjunct to standard therapy	Hospital admission and PEF.	120 patients (F: 77%). MgSO4: 58. Control: 62. Acute asthma. 18–65 Y.	SoC: nebulized albuterol, i.v. steroids, oxygen. MgSO4: 2 g i.v. infused over 20 min. Control:?	No effect of i.v. MgSO4. Hospital admission: MgSO4: 22%, CI: 13%–32%. Control: 17%, CI: 10%–26%, *p* = 0.523. ⊿PEF: MgSO4: 122 ± 75 L/min, CI 106–138 L/min. Control: 133 ± 82 L/min, CI: 116–150 L/min, *p* = 0.419.
Tiffany (12), 1993, Detroit, United States	RCT, double blind	I.v. MgSO4 (as a bolus and/or infusion) as adjunct to standard therapy	PEF and FEV_1_ (260 min)	48 patients (F: 56%). MgSO4 bolus: 15, MgSO4 infusion: 12, placebo: 21. Acute asthma (as defined by ATS-guidelines and initial PEF <200l/min) 18–60 Y	SoC: nebulized albuterol, i.v. steroids, aminophylline. (1) MgSO4: 2 g i.v. over 20 min followed by 2 g/h over 4h (2) MgSO4: 2 g over 20 min followed by placebo infusion (3) placebo: placebo loading + infusion.	No effect of i.v. MgSO4. FEV_1_: *p* = 0.96. PEF: *p* = 0.61.
Bloch (10), 1995, New York, United States	RCT, double blind	I.v. MgSO4 as adjunct to standard therapy	Hospital admission (4 h), FEV_1_ (120 min)	135 patients (F: 72%). MgSO4: 67, control: 68. Acute asthma (as defined by ATS guidelines and FEV_1_ <75%p.) 18–65 Y	SoC: nebulized albuterol, i.v. steroids (FEV_1_ < 40%). MgSO4: 2 g i.v. Placebo: saline i.v.	Effect on patients with severe exacerbations, not moderate. Hospital admission: MgSO4: 25.4%, control: 35.3%, *p* = 0.21. FEV_1_: MgSO4: 55%; control: 56%, *p* = 0.92. Subgroup-analysis: Severe (FEV_1_ <25%): Admission: MgSO4: 33.3% (7/21), control: 78.6% (11/14), *p* = 0.009. FEV_1_: 120 min: *p* = 0.014, 240 min: *p* = 0.026
Boonyavorakul (13), 2,000, Thailand	RCT, double blind	I.v. MgSO4 as adjunct to standard therapy	Hospital admission (240 min)	33 patients (F: 88%). MgSO4: 17, control: 16. Acute asthma (FISCHL score > 4) 15–65 Y	SoC: i.v. steroids, nebulized salbutamol, O2 if necessary. MgSO4: 2 g i.v. Control: sterile water	No effect of i.v. MgSO4. Hospital admission: MgSO4: 3 (17.65%) Control: 4 (25.00%) RR: 0.71, CI: 0.19–2.67
Porter (14), 2001, United States	RCT, double blind	I.v. MgSO4 as adjunct to standard therapy	1: PEF 2: hospital admission (60 min)	42 patients (F: 64%). MgSO4: 18, control: 24. Acute asthma (PEF <100 L/min or <25%p) 18–55 Y	SoC: i.v. steroids, nebulized albuterol, O2 if necessary. MgSO4: 2 g IV Control: saline	No effect of i.v. MgSO4. PEF: MgSO4: 211 ± 104 L/min, control: 252 ± 108 L/min, *p* = 0.073 Hospital admission: MgSO4: 5/18 (28%), control: 5/24 (21%). *p* = 0.72.
Silverman (15), 2002, United States	RCT, double blind	I.v. MgSO4 as adjunct to standard therapy	1: FEV_1_%p 2: hospital admission, PEF (240 min)	240 patients (120 + 120), (F: 42%) Acute asthma (FEV_1_ <30%p) 18–60 Y	SoC: i.v. steroids, nebulized albuterol, O2. MgSO4: 2 g i.v. Control: like-appearing placebo	Effect of i.v. MgSO4 on FEV_1_ and PEF. FEV_1_%p: MgSO4: 48.2%, control 43.5%, mean difference 4.7 (0.29–9.3), *p* = 0.045. PEF: MgSO4: 272 L/min, control: 236 L/min, mean difference 36 L/min (8–64 L/min), *p* < 0.01 No effect on hospital admissions: MgSO4: 39/122 = 32%, control: 41/126 = 32%
Bradshaw (16), 2007, United Kingdom	RCT, double blind	I.v. MgSO4 as adjunct to standard therapy	1: PEF%p 2: hospital admission (60 min)	129 patients (F: 57%). MgSO4: 62, control: 67. Acute asthma (PEF <75%p) >16 Y	SoC: 35% O2, i.v. steroids, nebulized salbutamol + ipratropium. MgSO4: 1.2 g i.v. Control: saline	No effect of i.v. MgSO4. PEF%p: MgSO4: 65.4, control: 62.8, mean difference 2.6 (−4.66–9.93), *p* = 0.48. Hospital admission: MgSO4: 49/62 = 79%, control: 52/67 = 78%, *p* = 0.98. Sub-group analysis: life-threatening (PEF ≤33%): No difference in PEF%p (*p* = 0.85) or hospital admission (*p* = 0.50)
Singh (8), 2008, India	RCT, single blind	I.v. MgSO4 as adjunct to standard therapy	FEV_1_%p, hospital admission (120 min)	60 patients (30 + 30), (F: 52%) Acute asthma (FEV_1_ <30%p) 18–60 Y	SoC: i.v. steroids, nebulized salbutamol + ipratropium. MgSO4: 2 g i.v. Control: saline	Effect of i.v. MgSO4. ΔFEV_1_%p: MgSO4: 40.77 ± 9.2% control: 34.7 ± 7.3%. Mean difference 6.07% (1.87–10.62) *p* < 0.01. Hospital admission: MgSO4: 2/30, control: 9/30, *p* = 0.011.
Goodacre (9), 2014, United Kingdom	RCT, double blind	I.v. or nebulized MgSO4 as adjunct to standard therapy	1: hospital admission 2: PEF	1,084 patients (F: 70%). i.v. MgSO4: 394, placebo: 358. Nebulized MgSO4: 332, placebo: 358. Acute asthma (PEF <50%p, RR >25, HR >110 or trouble talking) >16 Y	SoC: Differing (O2, steroids, salbutamol, ipratropium) MgSO4: 2 g i.v. Control: saline or MgSO4: Nebulized 500 mg MgSO4 × 3 Control: saline	No effect of i.v. or nebulized MgSO4. ΔPEF: I.v. MgSO4: 61.0 L/min, nebulized MgSO4: 58.3 L/min, control: 62.5 L/min. I.v. compared with placebo: *p* = 0.680. Nebulized compared to placebo: *p* = 0.664. Hospital admission: I.v. MgSO4: 285/394 = 72%. Nebulized MgSO4: 261/332 = 79%. Control: 281/358 = 78%. I.v. compared with control: *p* = 0.083, nebulized compared with control: *p* = 0.819.
Nannini (17), 2,000, Argentina	RCT, double blind	Nebulized MgSO4 as adjunct to salbutamol	1: PEF (20 min)	35 patients. (F: 33%) MgSO4: 19, control: 16. Acute asthma. >18 Y	SoC: nebulized salbutamol MgSO4: nebulized 3 ml MgSO4. Control: nebulized 3 ml saline.	Effect of nebulized MgSO4. ΔPEF: MgSO4: 134 ± 70 L/min, control: 86 ± 64 L/min, *p* = 0.05.
Bessmertny (18), 2002, United States	RCT, double blind	Nebulized MgSO4 as adjunct to albuterol	FEV_1_%p (125 min)	74 patients (37 + 37) (F: 73%). Acute asthma (PEF: 40%–80%p) 18–65 Y	SoC: nebulized albuterol + O^2^. MgSO4: nebulized 384 mg MgSO4 × 3 Control: saline Steroids for patients that did not respond to treatment	No effect of nebulized MgSO4. FEV_1_%p: MgSO4: 59, control: 64, CI: −4 to 14.
Hughes (7), 2003, New Zealand	RCT, double blind	Nebulized MgSO4 as adjunct to standard therapy	1: FEV_1_ 2: hospital admission (90 min)	52 patients (F: 52%). MgSO4: 28, control: 24. Acute asthma [FEV_1_ <50%p (after 2.5 mg salbutamol)] 16–65 Y	SoC: i.v. steroids, nebulized salbutamol. MgSO4: nebulized 151 mg MgSO4 × 3. Control: nebulized saline	Effect of nebulized MgSO4. FEV_1_: MgSO4: 1.96l, control: 1.55l. Mean difference: 0.37l (0.13–0.61, *p* = 0.003) Hospital admissions: MgSO4: 12/28, control: 17/24, RR 0.61 (CI: 0.37–0.99), *p* = 0.04
Kokturk (19), 2005, Türkiye	RCT, single blind	Nebulized MgSO4 as adjunct to standard therapy	1: PEF 2: hospital admission (240 min)	26 patients (F:73%). MgSO4: 14, control: 12. Moderate-to-severe asthma attack (GINA 2002 criteria) 18–60 Y	SoC: O2, steroids, nebulized salbutamol. MgSO4: nebulized isotonic MgSO4 (145 mg max x6) Control: saline	No effect of nebulized MgSO4. PEF: MgSO4: significant change at 120 min. Control: significant change at 60 min. Hospital admission: MgSO4: 1/14, control: 2/12
Gallegos-Solórzano (20), 2010, Mexico	RCT, double blind	Nebulized MgSO4 as adjunct to standard therapy	1: FEV_1_ 2: ED and hospital admission (90 min)	60 patients (30 + 30) (F: 70%). Acute asthma (FEV_1_ <60%p) >18 Y	SoC: i.v. steroids + nebulized albuterol + ipratropium. MgSO4: nebulized 333 mg MgSO4 × 3. Control: nebulized saline	Effect of nebulized MgSO4. FEV_1_%p: MgSO4: 69.7 ± 13.3, control: 61.13 ± 12.7, *p* < 0.01. ED admission: MgSO4: 5/30 = 16%, control: 13/30 = 43%, *p* = 0.04. Hospital admission: MgSO4: 2/30 = 7%, control: 7/30 = 23%, *p* > 0.05
Ahmed S (21), 2013, Bangladesh	RCT, open	To compare the efficacy of nebulized MgSO4 with salbutamol to normal saline and salbutamol in severe acute asthma	1: PEF (20 min)	120 patients. (60 + 60), (F:?) Severe acute asthma, not further elaborated	SoC: nebulized salbutamol (unknown dose) MgSO4: nebulized MgSO4 (unknown dose) Control: nebulized saline	Effect of nebulized MgSO4. PEF%p: Poor response (<40%): MgSO4: 14/60 = 23.3%, control: 41/60 = 68.3%. Incomplete response (40–69%): MgSO4: 41/60 = 68.3%, control: 19/60 = 31.7% Good response (≥70%): MgSO4: 5/60 = 8.3%, control: 0/60 = 0%. *p* < 0.001.
Badawy (22), 2014, Egypt	RCT	Nebulized MgSO4 as adjunct to standard therapy	1: FEV_1_, PEF (120 min)	60 patients (30 + 30) (F: 100%). Pregnant women with acute asthma. Adults (around 25 Y)	SoC: O2, i.v. steroids, i.v. aminophylline, nebulized salbutamol. MgSO4: nebulized 500 mg MgSO4 × 3 Control: saline	Effect of nebulized MgSO4. FEV_1_: MgSO4: 56.31 ± 8.25, control: 32.86 ± 7.15, *p* < 0.001. PEF: MgSO4: 54.20 ± 11.09, control: 36.17 ± 10.80, *p* < 0.001
Hossein (23), 2016, Iran	RCT, double blind	Nebulized MgSO4 as adjunct to standard therapy	1: PEF%p 2: hospital admission (60 min)	50 patients (25 + 25), (F: 50%) Acute asthma (PEF <60%p) >16 Y	SoC: oral steroids, nebulized salbutamol and ipratropium. MgSO4: cumulative 1.5 g. Control: saline	Effect of nebulized MgSO4. PEF%p: MgSO4: 48.7 ± 23.4, control: 36 ± 28.7, *p* = 0.002. Hospital admission: MgSO4: 11/25 = 44%, control: 18/25 = 72%, *p* = 0.02.

### Intravenous magnesium sulfate in acute refractory asthma exacerbation

Only three of the nine studies investigating treatment with i.v. MgSO4 for acute asthma exacerbation found a significant effect on PEF and/or FEV_1_ compared to placebo. Bloch et al. did not find a significant difference between groups when comparing all the patients included (135 patients, FEV_1_ <75%predicted), but when analyzing data for a subgroup of patients presenting with exacerbation and FEV_1_ <40%predicted (35 patients), they did find a significant effect of MgSO4 in this subgroup of patients (only *p*-values given: after 120 min: *p* = 0.014, 240 min: *p* = 0.026) (15). Singh et al. found that the group treated with i.v. MgSO4 had a higher FEV_1_%predicted (62.8%pred ± 10.0% vs. 56.7%pred ± 6.2%) and significantly greater %predicted improvement from baseline (40.7%pred ± 9.2% vs. 34.7%pred ± 7.3%, *p* < 0.01) compared to the placebo group (9). At the final assessment, Silverman et al. found that mean FEV_1_ in the i.v. MgSO4 group was 48.2%predicted compared to 43.5%predicted in the placebo group (mean difference 4.7%predicted; *p* = 0.045), and there was also a statistically significant improvement in PEF (mean difference 36 L/min; *p* = 0.01) (11).

In conclusion, three studies comprising a total of 335 patients found an effect of i.v. MgSO4 on improvement in PEF/FEV_1_ (although one of these studies only found an effect in a subgroup analysis) (9, 11, 15), whereas six studies with a total of 1,124 patients did not find any effect (10, 12–14, 16, 17).

Only two of the eight studies, which also investigated hospital admission, found that i.v. MgSO4 significantly increased the hospital discharge rate compared to placebo. Singh et al. showed that the discharge rate after two hours was significantly higher in the MgSO4 group (placebo: 21/30 and MgSO4: 28/30, *p* < 0.05) (9). Bloch et al. did not find an effect on the entire patient cohort, but in the subgroup analysis with patients suffering from severe exacerbations (FEV_1_ <25%predicted), they found a significant difference in hospital admission rates between the patients treated with MgSO4 (7/21 = 33.3%) compared with the placebo group (11/14 = 78.6%, *p* = 0.009) (15).

All the studies except one (13) also described side effects of i.v. MgSO4. The most common side effects were fatigue (14, 15) and flushing (14, 15, 17), but none of the studies observed severe adverse effects.

### Nebulized magnesium sulfate in acute refractory asthma exacerbation

Six of the nine studies investigating treatment with nebulized MgSO4 compared to placebo in acute asthma exacerbations found a significant effect on FEV_1_ and/or PEF compared to placebo. The study by Nannini et al. showed a greater increase in PEF in the MgSO4 group at 10 min after baseline (difference 30%; 95% CI: 3% to 56%; *p* = 0.03) and again at 20 min after baseline (difference 57%; 95% CI: 4% to 110%; *p* = 0.04) compared to the placebo group. The absolute increase, however, did not statistically differ significantly at any time point (10 min difference: 23 L/min, *p* = 0.18; 20 min difference: 48 L/min, *p* = 0.05) (20). Hughes et al. found a significant difference in the mean FEV_1_ between the two groups (0.37 L; *p* = 0.003) in favor of MgSO4 (8). Gallegos-Solórzano et al. showed that adding nebulized MgSO4 to the treatment resulted in statistically significant increases in FEV_1_%predicted (placebo: 61.13%pred ± 12.7 vs. MgSO4: 69.7%pred ± 13.3; *p* < 0.01) (21). Ahmed et al. found that the % increase in PEF after 20 min was significantly greater in the MgSO4 group (35% ± 7%) than in the placebo group (24% ± 6%, *p* < 0.001) (22). The study by Badawy et al. similarly found significant improvement of FEV_1_ after 120 min in the MgSO4 group compared to placebo (MgSO4: 56.31 ± 8.25, control: 32.86 ± 7.15, *p* < 0.001, measuring unit not given) (23). Hossein et al. showed that PEF%predicted was significantly higher in the MgSO4 group (48.7%pred ± 23.4) than in the placebo group (36%pred ± 28.7; *p* = 0.002 and *p* < 0.001) after 60 min (18).

Even though six studies found an effect of nebulized MgSO4 on PEF/FEV_1_, the studies only included a total number of 377 patients, compared to the 790 patients in the three studies that did not find an effect (10, 19, 24).

Only two of five studies, which also investigated hospital admission, found that nebulized MgSO4 increased the hospital discharge rate compared to placebo. Hughes et al. showed that the hospital admission rate was significantly higher in the placebo group (MgSO4: 12/28, placebo: 17/24; *p* = 0.04) (8). Hossein et al. found that the hospital admission rate after 60 min was lower in the MgSO4 group compared to placebo (MgSO4: 44%, placebo: 72%, *p* = 0.02) (18).

All studies besides that of Badawy (23) also described the difference in adverse effects between groups. The most common adverse effects of MgSO4 described are transient hypotension (10, 24) and bitter taste (19, 21), but none of the adverse effects resulted in withdrawal from the studies. Three studies did not report any adverse effects (8, 18, 20).

### Pooled estimate analysis of PEF measurements

A total of eight studies were included in the meta-analyses for a pooled estimate analysis of mean differences in PEF measurements. Four studies in one meta-analysis illustrated an effect of i.v. MgSO4 compared to placebo, and four studies in another meta-analysis illustrated nebulized MgSO4 compared to placebo. Excluded studies provided measurements of FEV_1_ and/or PEF%predicted and were hence not included in the meta-analyses. In total, 793 patients were treated with i.v. MgSO4, and 575 patients were treated with nebulized MgSO4. In all, 592 of those receiving i.v. MgSO4 and 441 of those receiving nebulized MgSO4 were included in the meta-analyses.

Through a pooled estimate analysis of mean differences, the weighted mean difference in PEF measurements using a random-effects model was 5.49 (95% CI, −18.67 to 29.65, *p* = 0.10) in patients receiving i.v. MgSO4 compared to placebo, while the weighted mean differences using a random-effects model was 23.57 (95% CI, −2.48 to 49.62, *p* < 0.01) in patients receiving nebulized MgSO4 compared to placebo.

The analysis of patients receiving i.v. MgSO4 compared to placebo was not significant, while the analysis of nebulized MgSO4 compared to placebo showed a statistically significant difference.

**Figure 2:**
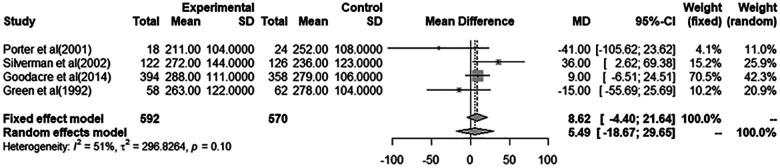
Meta-analysis using a random effects model to determine mean peak expiratory flow (L/min) difference (95% confidence interval) between placebo/controls and severe asthma patients receiving intravenous magnesium sulfate.

**Figure 3:**
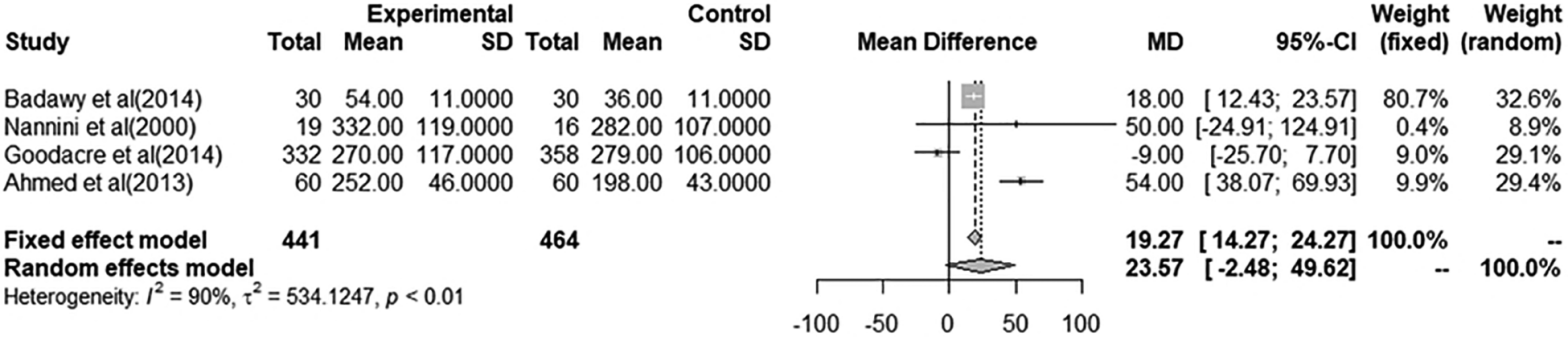
Meta-analysis using a random effects model to determine mean peak expiratory flow (L/min) difference (95% confidence interval) between placebo/controls and severe asthma patients receiving nebulized magnesium sulfate.

More details are provided in Figures 2, 3.

### Sensitivity analysis

The pooled effect size estimate analysis was repeated in series after stepwise omission of each included RCT in a sensitivity analysis, which revealed that no individual study had an impact of the mean difference estimate of more than 8.86 L/min in studies focusing on difference in PEF between i.v. and placebo (variation of estimates was −3.37 [−28.18–21.43] to 11.30 [−11.23–33.83]) and 13.91 L/min in studies focusing on difference in PEF between nebulized and placebo (variation of estimates was 9.66 [−14.72–34.03] to 37.06 [5.29–68.83]) (Table 2).

**Table 2:** Sensitivity analysis of included studies focusing on either (A) intravenous or (B) nebulized magnesium sulfate.

Randomized controlled trial omitted	MD (95% CI)	Heterogeneity
(A)		
Porter et al. (16)	11.30 (−11.23–33.83)	*I*^2^ = 47%, *t*^2^ = 193.8484, *p* = 0.15
Silverman et al. (11)	−3.37 (−28.18–21.43)	*I*^2^ = 35%, *t*^2^ = 195.7486, *p* = 0.21
Goodacre et al. (10)	−1.27 (−46.21–43.68)	*I*^2^ = 67%, *t*^2^ = 1040.1831, *p* = 0.05
Green et al. (12)	10.42 (−18.95–39.78)	*I*^2^ = 57%, *t*^2^ = 377.1589, *p* = 0.10
Total	5.49 (−18.67–29.65)	*I*^2^ = 51%, *t*^2^ = 296.8264, *p* = 0.10
(B)		
Nannini et al. (20)	21.00 (−6.74–48.74)	*I*^2^ = 93%, *t*^2^ = 553.4887, *p* < 0.01
Ahmed et al. (22)	9.66 (−14.72–34.03)	*I*^2^ = 80%, *t*^2^ = 301.8074, *p* < 0.01
Goodacre et al. (10)	37.06 (5.29–68.83)	*I*^2^ = 89%, *t*^2^ = 567.8665, *p* < 0.01
Badawy et al. (23)	28.62 (−23.63–80.87)	*I*^2^ = 93%, *t*^2^ = 1755.0169, *p* < 0.01
Total	23.57 (−2.48–49.62)	*I*^2^ = 90%, *t*^2^ = 534.1247, *p* < 0.01

### Quality and bias risk assessment

Overall, the studies on i.v. magnesium sulfate for acute asthma had a low risk of bias. Boonyavorakul, Silverman, and Goodacre were classified as having low risk of bias in all categories. See Table 3 for the total assessment.

**Table 3:** Risk of bias analysis of the studies on IV magnesium sulfate for acute asthma.

Study	Risk of bias from the randomization process	Risk of bias due to deviations from the intended interventions	Risk of bias due to missing outcome data	Risk of bias in measurement of the outcome	Risk of bias in selection of the reported results	Overall risk of bias
Green	High risk	High risk	Low risk	Some risk	Some risk	High risk
Tiffany	Some risk	Low risk	Low risk	High risk	High risk	High risk
Bloch	Low risk	Low risk	Low risk	Low risk	High risk	High risk
Boonyavorakul	Low risk	Low risk	Low risk	Low risk	Low risk	Low risk
Porter	Low risk	Low risk	Low risk	Low risk	Some risk	Low risk
Silverman	Low risk	Low risk	Low risk	Low risk	Low risk	Low risk
Bradshaw	Low risk	Low risk	Low risk	Low risk	Some risk	Some concerns
Singh	Low risk	Low risk	Some risk	Low risk	Low risk	Low risk
Goodacre	Low risk	Low risk	Low risk	Low risk	Low risk	Low risk

The studies on nebulized magnesium sulfate for the treatment of acute asthma generally had a higher risk of bias. Of the nine studies included, only Goodacre was low in risk of bias in all categories. Many of the studies had a high risk of bias in their reporting of results, mostly because data were not reported on all the outcomes of interest (see Table 4).

**Table 4:** Risk of bias analysis of the studies on nebulized magnesium sulfate for acute asthma.

Study	Risk of bias from the randomization process	Risk of bias due to deviations from the intended interventions	Risk of bias due to missing outcome data	Risk of bias in measurement of the outcome	Risk of bias in selection of the reported results	Overall risk of bias
Nannini	Some risk	Low risk	High risk	Low risk	High risk	High risk
Bessmertny	Low risk	Low risk	Some risk	Low risk	High risk	High risk
Hughes	Low risk	Low risk	Low risk	Low risk	High risk	High risk
Kokturk	Some risk	Some risk	Low risk	High risk	Low risk	High risk
Gallegos-Solórzano	Some risk	Low risk	Low risk	Low risk	Low risk	Some concerns
Ahmed	Some risk	High risk	High risk	High risk	High risk	High risk
Goodacre	Low risk	Low risk	Low risk	Low risk	Low risk	Low risk
Badawy	Some risk	High risk	High risk	Low risk	High risk	High risk
Hossein	Low risk	Low risk	Some risk	Low risk	Some risk	Some concerns

## Discussion

The evidence concerning treatment of acute asthma with MgSO4 continues to be, as shown above, rather conflicting, irrespective of route of administration of MgSO4.

Nine RCTs investigate the effect of i.v. MgSO4 on acute asthma, three studies demonstrate an effect of i.v. MgSO4 (9, 11, 15) (335 patients), and six studies do not find a significant effect of i.v. MgSO4 as an add-on to standard therapy (10, 12–17) (1,124 patients).

The MgSO4 dose administered ranges from 2 g (9, 11, 14, 16) to 1.2 g (17), and no obvious relationship between the size of dose and effect on outcome is seen.

If we look at the RCTs' different measurements used to determine the effect of MgSO4, there is no clear association either. Objective measurements of lung function as FEV_1_ and PEF are used by studies that find an effect of MgSO4 (9, 11, 15) and by studies that do not find an effect (10, 13, 16, 17).

A more subjective measure used is hospital admission rate, which is very relevant because it is a measure that directly affects the patient (who will either spend days in the hospital or go home) and the economics of the healthcare system. Two studies (9, 15) (only sub-analysis)) find a decrease in hospital admission in the MgSO4 group compared to placebo, and six studies (10–12, 14, 16, 17) do not find any difference between the two groups.

Another possible explanation for the conflicting results could be differences in severity of asthma exacerbations, but if we assume that FEV_1_ and PEF are comparable measurements, the results concerning severe exacerbations are conflicting and not exclusively pointing towards an effect of MgSO4 as implied (9, 11, 15).

When only looking at the studies with low risk of bias, the results are still conflicting. When excluding Green (12), Bradshaw (17), Bloch (15), and Tiffany (13) from the analysis, two studies find an effect of MgSO4 on lung function (*n* = 200) and three do not (*n* = 827).

In the nine RCTs investigating the effect of nebulized MgSO4 in acute asthma, six studies find a significant effect of MgSO4 (*n* = 377), while three studies do not (*n* = 790). The conflicting results regarding nebulization cannot be explained by the size of the MgSO4 dose given or by the different outcome measurements either. The treatment dose of MgSO4 differs between the different studies. Three studies give the highest cumulative dose of 1,5 g of nebulized MgSO4 (10, 18, 23). Of these three studies, two find an effect, and one does not find an effect of MgSO4, which indicates that the difference in the given dose is unlikely to explain the different results.

The study with the second highest MgSO4 dosage is that of Bessmertny et al. (384 mg × 3) (19), who do not find an effect of MgSO4 either.

When delivering medication through a nebulizer compared to i.v., the delivered dose also depends on, among other factors, particle size and device technique. Most of the studies provide details on administering isotonic MgSO4, and some also describe the specific nebulizer used (jet nebulizer (8, 20, 24) or circulaire nebulizer (19)), but it is not possible to assess the impact of the more precise impact on particle sizes and delivered dose in the included studies.

Four studies use FEV_1_ (8, 19, 21, 23) as the outcome measure, of which three find a significant effect of MgSO4, and one study does not find any effect. The five other studies use PEF as a primary outcome; three find an effect (18, 20, 22) and two do not (10, 24).

Two studies find a significant decrease in hospital admission rate using MgSO4 (8, 18); three studies find no difference in admission rate (10, 21, 24).

Only Goodacre (10), who does not find an effect of nebulized MgSO4, has a low risk of bias in all categories. Gallegos-Solórzano (21) and Hossein (18) have some concerns in the analysis, while the rest of the studies reach high risk in at least one category. Gallegos-Solórzano and Hossein both find an effect of nebulized MgSO4, which results in conflicting results, even when taking the risk of bias analysis into account.

The Danish Society for Respiratory Medicine recommends treatment with i.v. MgSO4 in severe asthma exacerbation (3), with reference to MgSO4 having a proven effect on the length of hospital stay but not on the risk of need for intubation. This review, however, does not provide evidence that MgSO4 shortens hospital stays of asthmatic patients. Only two of the studies on i.v. MgSO4 find a significant effect of MgSO4 on discharge rate. Six studies do not find an effect of i.v. MgSO4 on hospital admission rate/length of stay.

The GINA guidelines from 2023 do not recommend the routine use of MgSO4 for asthma exacerbations but mention the possible effect in some patients suffering from severe exacerbations not responding well to standard treatment (4). Again, they recommend i.v. and not nebulized, MgSO4.

This recommendation fits better with the finding in our study, namely, not promising an evidential effect but using MgSO4 when proven treatments have been given without satisfying effect. The recommended pathway of delivering MgSO4 is, however, questionable.

The recommendation to give MgSO4 for patients not responding to standard treatment makes sense, considering that none of the studies included in this review report serious side effects to the treatment with MgSO4.

The findings reported for i.v. MgSO4 are not totally aligned with the latest Cochrane review performed on the same subject (1). Kew et al.'s review concludes that i.v. MgSO4 given to patients with status asthmaticus lowers hospital admission rate and improves lung function. Even though the Cochrane review was performed in 2014, no new RCTs have been included in this study that was not included in that review. The different conclusion may be caused by Kew et al. having included studies on children and studies where only abstracts were available. We decided to concentrate on adults since the way treatment works on children and adults is not always the same. We decided not to include studies that only published an abstract since we do not think an abstract provides enough information about the study for us to decide if the results are reliable.

Our conclusions on the doubtful effect of nebulized MgSO4 are very similar to those of the Cochrane review by Knightly et al. (25), even though they included studies on children and studies in which only abstracts were available.

The comparison of the different RCT's on the subject has not been easy. The SoC differs a lot between the studies; only five studies give nebulized SAMA; three studies do not use steroids at all, while two studies only include steroids if needed; and one study does not even add SABA to all the patients' treatments. This means that a lot of the included RCTs do not follow standard treatment guidelines for patients presenting with acute asthma. The RCTs not following standard treatment guidelines are represented in cases both for and against MgSO4. Bradshaw (17) and Goodacre (10) find no effect of i.v. MgSO4, while Singh (9) does find an effect; meanwhile, Gallegos-Solórzano (21) and Hossein (18) find an effect of nebulized MgSO4, while Goodacre (10) does not.

Another limitation worth mentioning is that the proportion of patients admitted differs between the included studies. In some studies, relatively few participants were included. This may be due to regional differences in hospital practices and varied clinical assessments of exacerbation severity. In addition, the included studies may have included individuals with a variety of differences in terms of gender, age, and ethnicity. These variations are usually adjusted for in the randomization process in each study. However, in our meta-analysis, it caused a high variation in our summary of mean differences. This is indicated by the higher *I*^2^ statistic in both the analysis of IV MgSO4 compared to placebo and nebulized MgSO4 compared to placebo, suggesting substantial heterogeneity.

## Conclusion

In conclusion, the reported findings regarding the treatment of acute asthma with intravenous/nebulized MgSO4 are conflicting. Overall, the evidence points against MgSO4 having a beneficial effect on lung function and decreasing admission rate in patients presenting with acute asthma. On the other hand, none of the included studies demonstrate severe side effects of MgSO4; thus, considering the low risk, treatment with MgSO4 can be attempted as a last resort in patients with refractory symptoms after standard treatment.

## Data Availability

The original contributions presented in the study are included in the article/Supplementary Material, further inquiries can be directed to the corresponding author.

## Key messages

• Of the studies investigating the effect of intravenous magnesium sulfate, more than half found no effect on lung function.
• Only two of the five studies investigating the effect of nebulized magnesium sulfate on hospital admission found a positive effect.
• The effect of intravenous or nebulized magnesium sulfate for acute asthma exacerbation refractory to standard of care treatment is inconclusive when assessed by lung function and hospital admission.
• The meta-analysis revealed a significant effect on PEF in asthma patients receiving nebulized MgSO4 compared to placebo, rather than an effect of intravenous MgSO4 compared to placebo, which was insignificant.
